# Supplementary material for: Random fractal-enabled physical unclonable functions with dynamic AI authentication
Source: Nat Commun. 2023 Apr 17;14:2185. doi: 10.1038/s41467-023-37588-5 (PMC10110537; doi:10.1038/s41467-023-37588-5)
Supplement: Supplementary file 1 — Supplementary Information [file 41467_2023_37588_MOESM1_ESM.pdf]

Supplementary Information for

Random fractal-enabled physical unclonable functions with dynamic AI authentication

Ningfei Sun[1,2], Ziyu Chen[1], Yanke Wang[3], Shu Wang[2], Yong Xie[1,4]* & Qian Liu[2]*

[1] School of Physics, Beihang University, Beijing 100191, China;

[2] CAS Center for Excellence in Nanoscience, National Center for Nanoscience and Technology & University of Chinese Academy of Sciences, Beijing 100190, China;

[3] Institute for Automation and Applied Informatics, Karlsruhe Institute of Technology, Karlsruhe 76344, Germany;

[4] Key Laboratory of Intelligent Systems and Equipment Electromagnetic Environment Effect, School of Electronic and Information Engineering, Beihang University, Beijing 100191, China;

*Corresponding author: Yong Xie, xiey@buaa.edu.cn; Qian Liu, liuq@nanoctr.cn

Table of Contents

Supplementary Note 1: Calculation of the encoding capacity 4

Supplementary Note 2: Calculation of the overall cost of a single PUF label 5

Supplementary Note 3: The ResNet50 based classification method 6

Supplementary Figure 1: Optical microscopy images of the surface morphological evolution process of 70 nm-thick Au film as a function of annealing time. 8

Supplementary Figure 2: 3D atomic force microscope image of the Au network structure with rugged surface 9

Supplementary Figure 3: X-ray diffraction (XRD) patterns of the Au film before and after the annealing process 10

Supplementary Figure 4: Optical microscope images of the network-based PUF tags from three different production batches 11

Supplementary Figure 5: Fractal dimension calculations of different network patterns 12

Supplementary Figure 6: Optical microscopy images of the PUF tags with different areas 13

Supplementary Figure 7: Heat map and the corresponding histogram distribution of the cross-correlation values 14

Supplementary Figure 8: Calculation of practical grayscale intensity level of the original PUF image 15

Supplementary Figure 9: Grayscale image (750*750) for the calculation of the encoding capacity 16

Supplementary Figure 10: Extraction of the network tag 17

Supplementary Figure 11: SEM images of the network surface morphology before and after the surface roughening process 18

Supplementary Figure 12: FDTD simulation of the roughened surface of the Au network 19

Supplementary Figure 13: SERS spectra contrast of R6G molecules on the network before and after the surface roughening 20

Supplementary Figure 14: Stability test under low temperature.. 21

Supplementary Figure 15: Stability test under high temperature 22

Supplementary Figure 16: Stability test under aqueous corrosion and mechanical friction....... 23

Supplementary Figure 17: Stability test under environmental contamination............................. 24

Supplementary Figure 18: Raman spectra of the same sample with the six-months interval..... 25

Supplementary Figure 19: Schematic illustration and Optical images presenting mass production of the PUF tags 26

Supplementary Figure 20: The grayscale distribution before and after the image preprocessing of grayscale stretch.. 27

Supplementary Figure 21: The pipeline of the developed PUF key authentication software..... 28

Supplementary Figure 22: Demonstration of PUF readout 29

Supplementary Figure 23: The 600 images as a part of the PUF key database for training/validation of the deep learning model... 30

Supplementary Figure 24: Classification accuracy increased with CNN DL process................. 33

Supplementary Figure 25: Three kinds of images from the test-set.. 34

Supplementary Figure 26: The preprocessing of the uploaded image 35

Supplementary Table 1: The dataset information for the AI model training/validation/testing.. 36

Supplementary References... 37

Supplementary Note 1. Calculation of the encoding capacity

According to the general binary-bit model established by Carro-Temboury et al[1], the encoding capacity can be expressed as follows,

$$\#\mathrm{Code} = \left[C \left(1 + L \left(\frac{1}{\sqrt{D}} - 1 \right) \right)^2 + 1 \right]^{D \frac{R^2}{L^2}} \tag{1}$$

where C is the number of colors (i.e., levels of grayscale intensity) of the network pattern, L is the length of each unit, D is the pattern filling density, and R is the resolution. In Supplementary Figure 9, the image size of the network tag is assumed to be 750×750 for simplifying the calculation process. Each single image is divided into 30×30 arrays, where each array unit is further divided into 5×5 subunit arrays (i.e., Length of each unit, L = 5; Resolution, R = 150). The theorical grayscale intensity of each pixel is in the range of 0-255. Considering the practical imaging condition and the image contrast, C is considered as 140 by subtracting the invalid grayscale information (Supplementary Figure 8). The physical features of one network pattern can be extracted with the threshold of 127 via ImageJ software and the pattern filling ratio (D) is regarded as the proportion of the network pattern to the whole image. Thus, we used a D value of 0.088 as an example to calculate the encoding capacity. According to equation 1, the encoding capacity of the PUF pattern in Supplementary Figure 9 is estimated to be 10^{348} >> 10^{20}, which is the basic PUF key encoding capacity[2]. Note that if a security label is composed of multiple PUF tags, the encoding capacity will be even higher (theoretically reaching 10^{348n}, where n is the number of the PUF tags involved).

Supplementary Note 2. Calculation of the overall cost of a single PUF label.

Material cost:

Cost of 1g Au: US$ 53 (the value is the average international price of gold over the past five years)

The density of Au is 19.32 g/cm^3. For the convenience of product packaging, it is assumed that there is one PUF tag as the label per mm^2 and an area of 64 cm^2 on a 4-inch wafer that can contain 6400 PUF tags. The volume of the 70 nm-thick Au film on an area of 1 cm^2 is 7×10^{-6} cm^3. As the density of Au is 19.32 g/cm^3, the weight of Au on one wafer is 8.66×10^{-3} g and the cost of one wafer is US$ 0.46. The Au cost required for one label is, therefore, about US$ 7×10^{-5}.

Cost of one piece of 4-inch wafer: US$ 22.

The cost of wafers for each label is about US$ 0.0034. It's assumed that five wafers composed of tags can be produced per day. 300 days are taken to fabricate the labels per year. About 9.6×10^7 labels can be fabricated in 10 years, and 15000 wafers are used.

Instrument costs:

Here only the main, large-scale machines are counted as follows,

1) Ultraviolet lithography machine (MJB4, SUSS MicroTec): US$ 147300
2) Magnetron sputtering machine (Kurt J. Lesker PVD75): US$ 176760
3) Muffle furnace (QSX1200): US$ 3093
4) Plasma cleaner (HM-Plasma5L): US$ 4124

These machines can be used for ten years or even longer. If we assume that 9.6×10^7 labels can be fabricated by these machines in 10 years, the average instrument cost for each security label is about US$ 0.0035.

Finally, the overall cost of one PUF label is estimated to be about US$ 0.0070 according to our desirable calculation strategy. The cost of the Au materials for the labels can be nearly ignored, which is attributed to the characteristic of mass production of our developed technique.

Supplementary Note 3. The ResNet50 based classification method

As each security code is unique to the others and is considered as one single class, we proposed a dynamic security code database strategy by using a ResNet50 based classification method. In order to generate more general and robust representation for each PUF pattern, a pretrained ResNet50 on ImageNet is modified (with an added 7×7 convolutional layer at the head and two fully connected layers at the end) as the classifier. The cross-entropy loss was used as the objective function of the classifier for multi-class classification, as follows,

$$\mathcal{L}(S,T) = -\frac{1}{C}\sum_{c=1}^{C} t_c \log s_c \quad (3)$$

where S is the softmax output (match score) generated by the classifier, T is the target label for each class, and C is the number of classes. In addition, the classifier can only generate the probability of each class instead of a certain label, so a set of predicted classes l for the input feature were obtained by

$$l = \{l_k\}_{k=1}^{K} = \underset{topK}{argmax}\langle S\rangle \quad (4)$$

where $l = \{l_k\}_{k=1}^{K}$ denotes the top possible K labels of the input feature, K=5 here, and $argmax_{topK}$ selects the indexes of the K largest values of S.

In order to avoid training a separate model for each pattern, we proposed a dynamic database strategy, where only one neural parameter (in the last layer of the classifier) was necessary to be added for a new PUF pattern. In addition, to reduce time consumption during the training procedure, only the parameters of the last layer for the classifier were trained when a new PUF pattern is added. Noting that both the original PUFs in the database and newly added PUFs are used to update the model to avoid the model forgetting the previous database.

Finally, the group of the predicted classes l were post-processed with a similarity-based method to get a higher accuracy (using the grayscale images of the input PUFs and target PUFs). The similarity $Sim_k(I_k, T_k)$ between the input grayscale image I_k and the target grayscale image T_k was computed by the maximum FSIM similarity value of I_k and rotated T_k.

Based on the above method, the feedback (F) can be sent to the client as follows,

$$F = \begin{cases} l_{k_m} & if\ sim_{k_m} > T_s \\ Please\ retry & otherwise \end{cases}, \tag{5}$$

where $k_m = argmax_k \langle \{Sim_k\}_{k=1}^{K} \rangle$,

$$sim_{k_m} = max_k \langle \{Sim_k\}_{k=1}^{K} \rangle$$

where T_s is the threshold of the similarity. If the maximum similarity is lower than the threshold T_s, the captured image from the client is not in the database, and then the client is required to capture another image with a better quality.

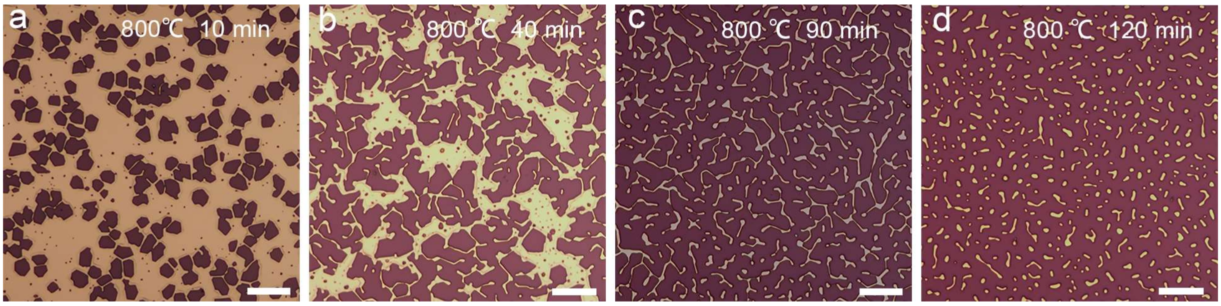

Supplementary Figure 1. Optical microscopy images of the surface morphological evolution process of 70 nm-thick Au film as a function of annealing time. a-d The corresponding optical microscopy images with different annealing times. A lower temperature is not enough for the formation of the voids and the further film shrinkage. Higher temperature can directly cause the formation of the spheroidized Au particles, which is not suitable for the use of the PUFs in our system due to its low complexity. Further annealing above 120 min can lead to the shrinkage and recrystallization of the irregularly shaped Au islands. Annealing time of 90 min is not a rigid parameter. A flexible change within a certain range of around 90 min can also lead to a satisfying network structure, probably with a slight difference in network length, which is not sufficient to influence the complexity and encoding performance of the network tag. Scale bars: 20 μm.

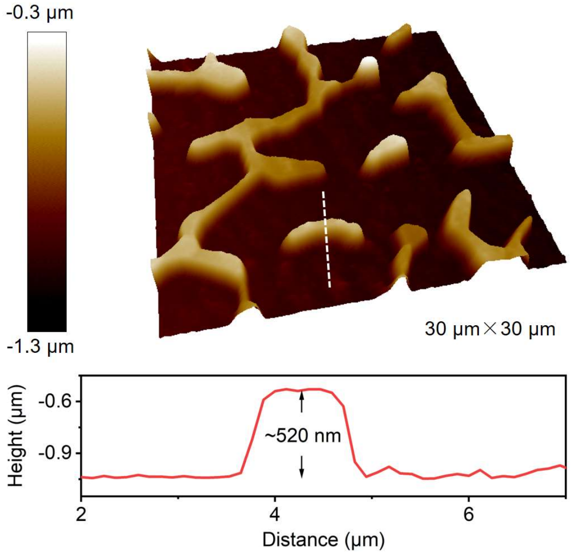

Supplementary Figure 2. 3D atomic force microscope image of the Au network structure with rugged surface.

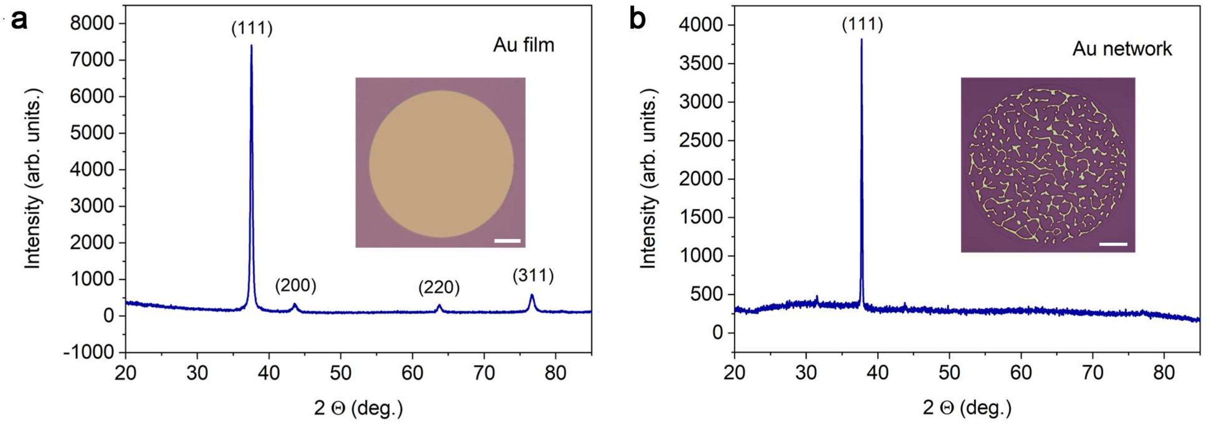

Supplementary Figure 3. X-ray diffraction (XRD) patterns of the Au film before and after the annealing process. a XRD pattern of the unannealed Au film and the corresponding optical image. **b** XRD pattern of the annealed Au network and the corresponding optical image, showing the evolution of the crystallinity from the polycrystalline structure of the Au film to the approximately single-crystalline structure of the Au network. Scale bars: 20 μm.

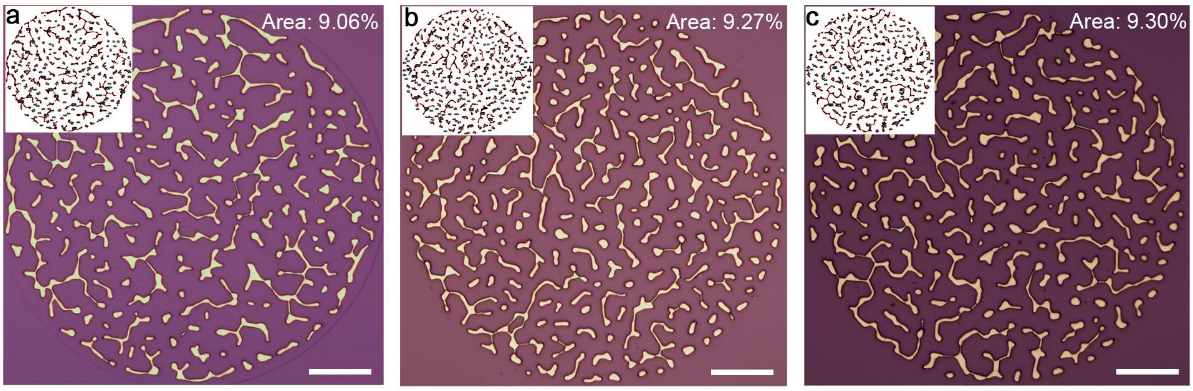

Supplementary Figure 4. Optical microscope images of the network-based PUF tags from three different production batches (a-c) under the same fabrication conditions (film thickness, annealing temperature, and annealing time). Either the visual inspection of the pattern morphology or the structure filling ratio calculated by ImageJ software can prove that the fabrication process of the Au networks has high reproducibility. Scale bars: 20 μm.

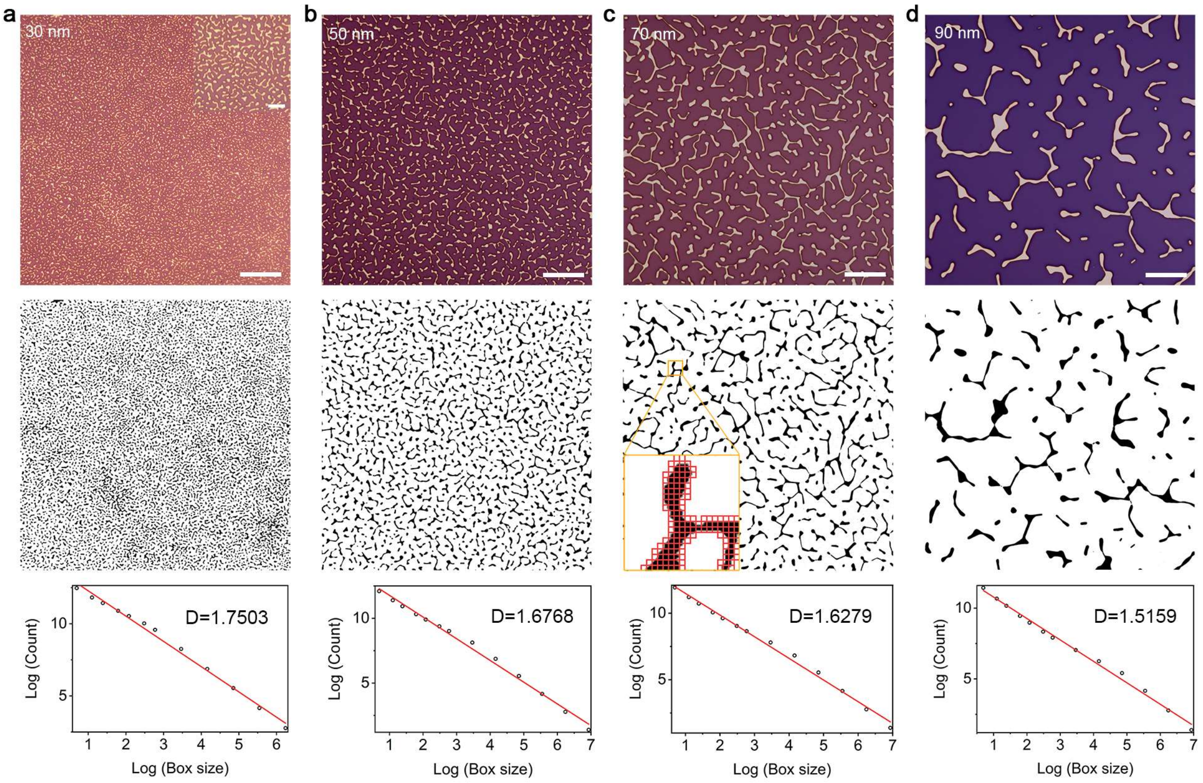

Supplementary Figure 5. Fractal dimension calculations of different network patterns. a-d Box-counting method was used to count the fractal dimension of the annealed Au network pattern from the Au film with thicknesses of 30 nm, 50 nm, 70 nm, and 90 nm. The fractal structure was first binarized and covered by the boxes, as shown in **c**, followed by reducing the area of the box exponentially. The fractal dimension D was finally obtained by calculating the slope based on linear regression as follows,

$$D = -\lim_{r \to 0} \frac{\log N(r)}{\log r} \tag{6}$$

where D is the fractal dimension, N is the number of the boxes, and r is the side of the original box. The fractal dimension can be directly obtained by ImageJ software with the FracLac plugin. Scale bars: 5 μm (**a**, inset), 20 μm (**a-d**).

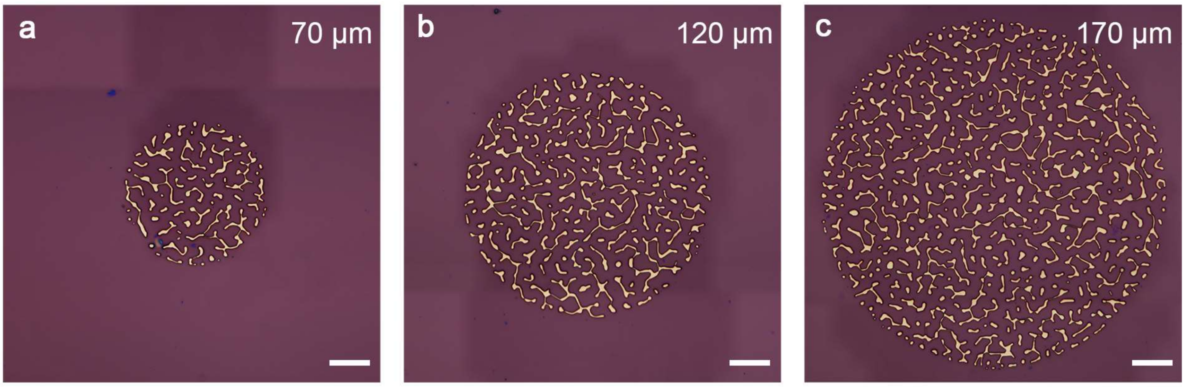

Supplementary Figure 6. Optical microscopy images of the PUF tags with different areas (a-c). The security level (i.e., encoding capacity) is proportional to the area of the PUF tag. Scale bars: 20 μm.

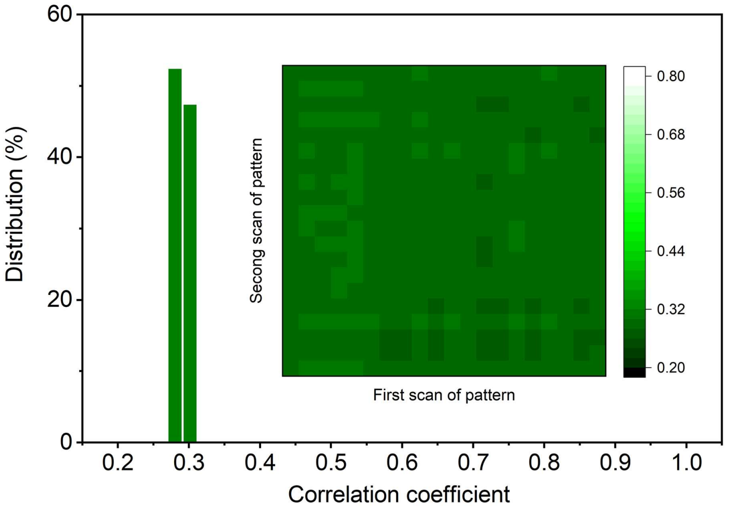

Supplementary Figure 7. Heat map and the corresponding histogram distribution of the cross-correlation values obtained from 40 network patterns in different fabrication batches.

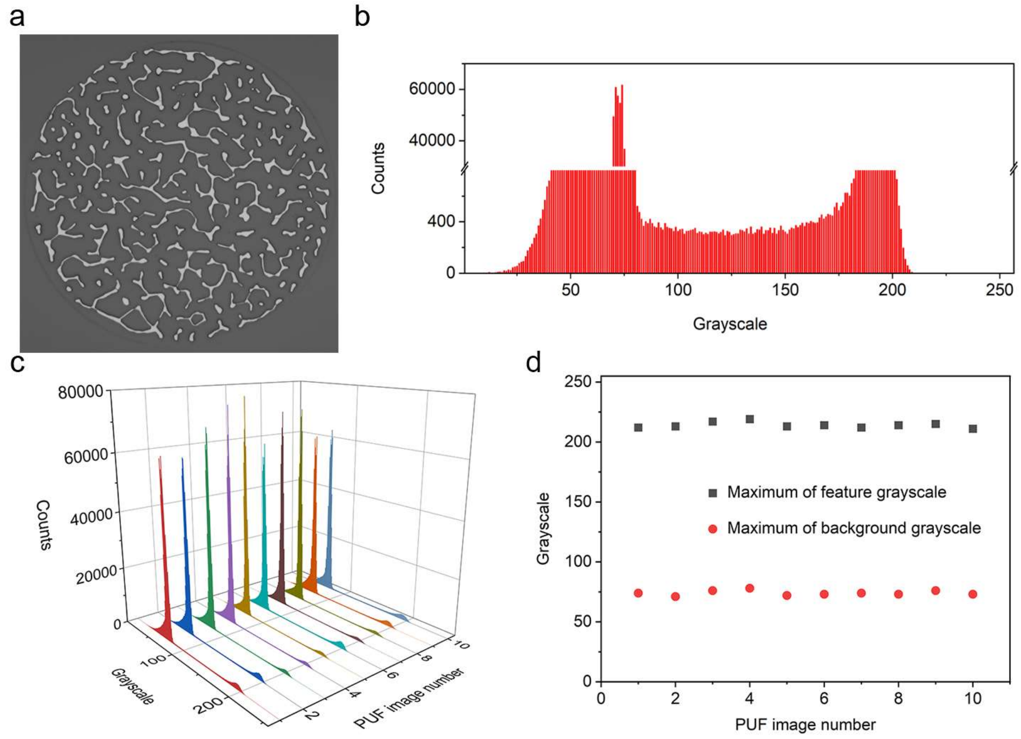

Supplementary Figure 8. Calculation of practical grayscale intensity level of the original PUF image. a Grayscale image of a typical PUF pattern. **b** Grayscale distribution histogram of a typical PUF pattern. **c** Grayscale distributions from 10 randomly selected PUF images in the basic database. **d** Maximums of feature grayscale and background grayscale from 10 PUF images in **c**. We defined the peak of grayscale histogram as the maximum of background grayscale, which means that the background loads the largest number of pixels with consistent grayscale intensity and the above grayscale intensities are all from the PUF feature structures. After subtracting the invalid grayscale information from background and the high grayscale intensities that the practical images cannot reach, we defined the grayscale range of the feature structures from 74 (average maximum of background grayscale) to 214 (average maximum of feature grayscale), and the level of grayscale is set as the difference, i.e., 140.

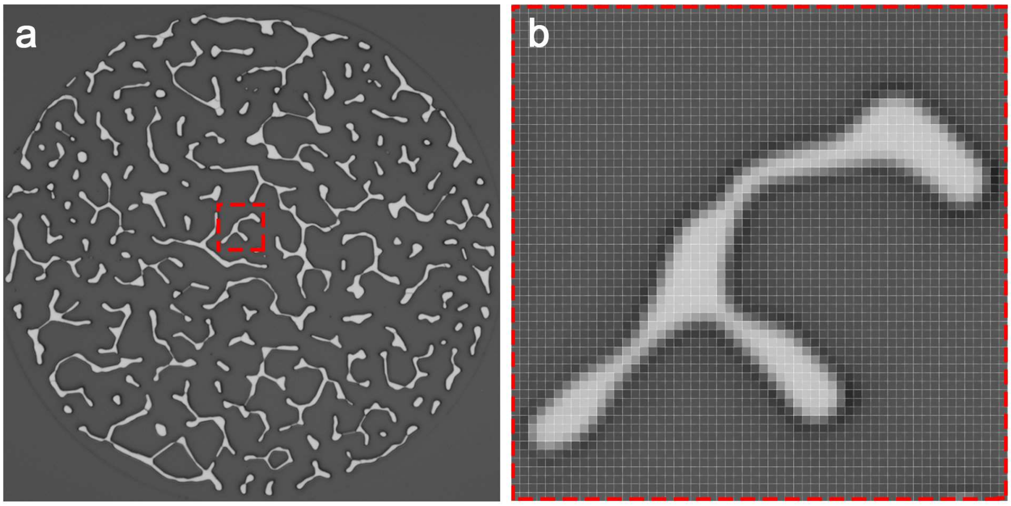

Supplementary Figure 9. Grayscale image (750*750) for the calculation of the encoding capacity. a The physical features extracted by Otsu's method. **b** The magnified grayscale image in the red region, showing the physical feature composed of single pixels. Every single pixel has a grayscale in the range of 74-214.

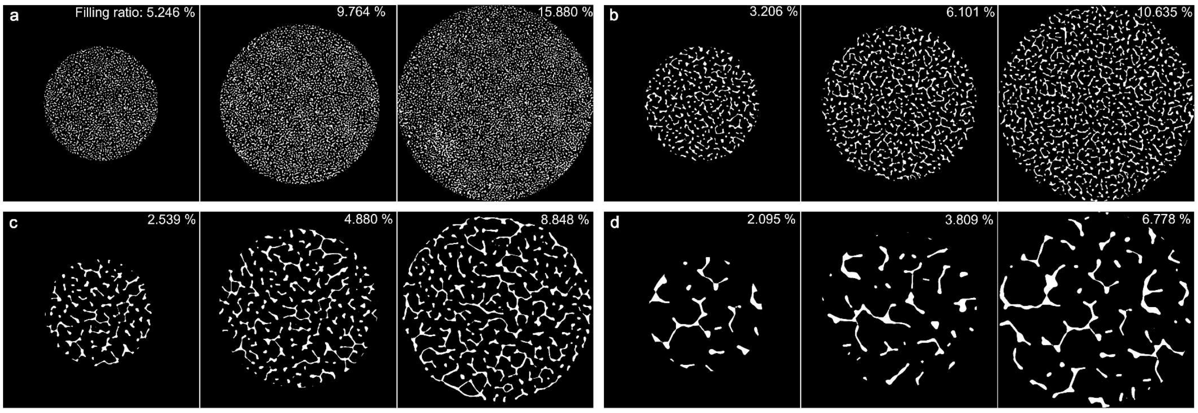

Supplementary Figure 10. Extraction of the network tag. a Images of the network tag derived from 30 nm-thick Au film, with a circular pattern diameter of 70 μm (Left), 95 μm (Medium), and 120 μm (Right), respectively. **b-d** The network patterns from the Au film with a thickness of 50 nm, 70 nm, and 90 nm, respectively. The physical features are extracted by ImageJ software and the corresponding network filling ratios shown in the images are calculated. The encoding capacity of the PUF pattern is mainly dependent on the feature filling ratio and pattern area, which can be flexibly regulated by the thickness of the Au film. The detailed calculations are shown in Supplementary Note 1.

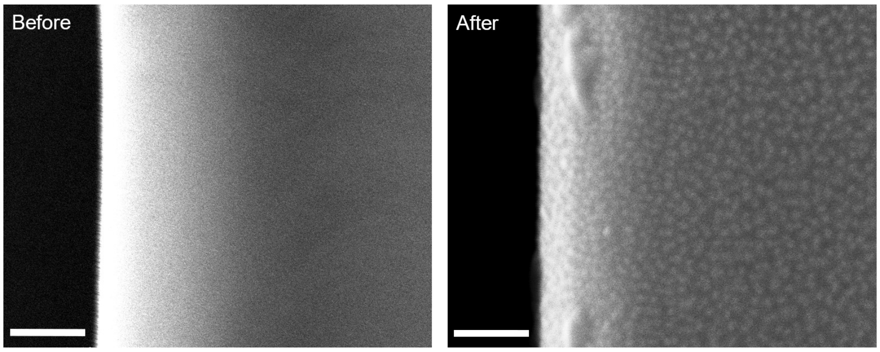

Supplementary Figure 11. SEM images of the network surface morphology before and after the surface roughening process, where the uniform surface convex nanostructures emerge on the surface. Scale bars: 100 nm.

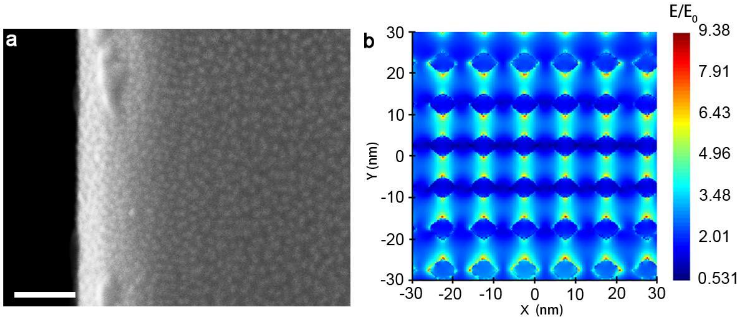

Supplementary Figure 12. FDTD simulation of the roughened surface of the Au network. a SEM image of the surface morphology of the Au network after the oxygen ion cleaning. Scale bar: 100 nm. **b** The electromagnetic-field "hot spots" distribution of the roughened surface at 514 nm excitation of light by using an equivalent Au array model that is approximate to actual surface morphology. Surface electrons in resonance under light excitation can result in an enhanced electromagnetic field in the close vicinity of the Au nanostructure surface.

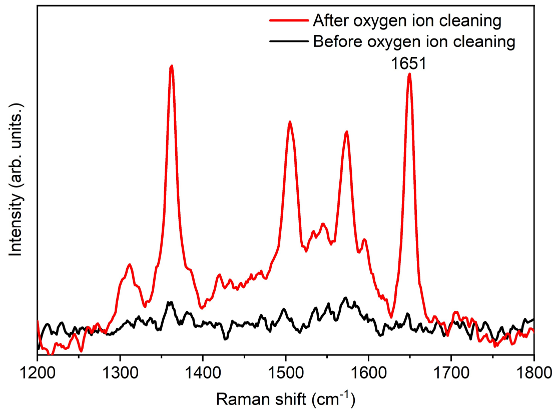

Supplementary Figure 13. SERS spectra contrast of R6G molecules on the network before and after the surface roughening. Due to the poor SERS effect of the flat surface of Au network, no Raman characteristic peaks can be observed.

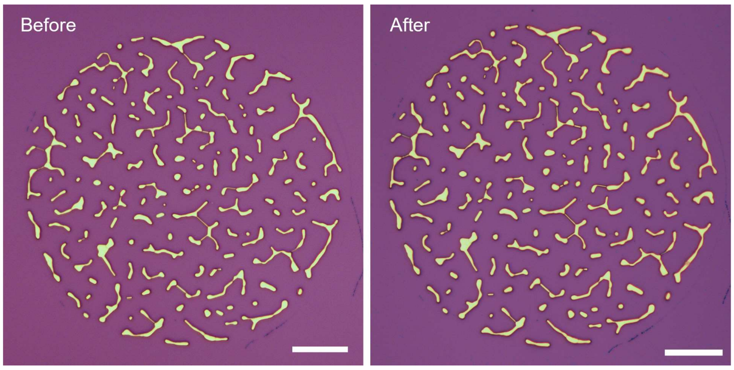

Supplementary Figure 14. Stability test under low temperature. The PUF label was refrigerated at -40 °C in the atmosphere for 60 hours, showing no variation of networks in topographical features. Scale bars: 20 μm.

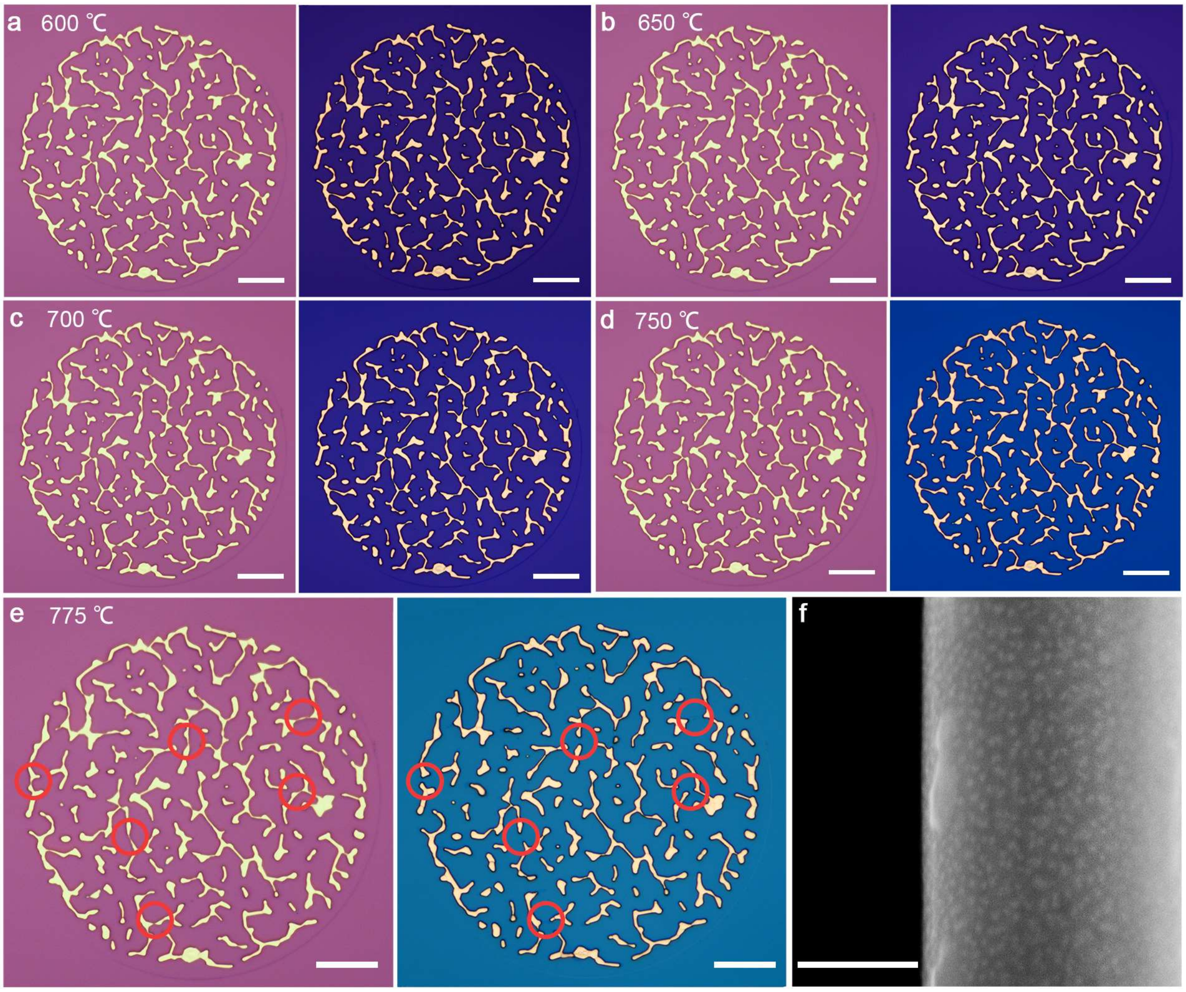

Supplementary Figure 15. Stability test under high temperature. a-d The surface morphology contrasts of one network tag under 600 °C, 650 °C, 700 °C, and 750 °C, respectively, showing no variation of the physical features. **e** The morphology contrast of the network tag under 775 °C. Slight changes of the network features can be observed as shown in the red circle. **f** The enlarged SEM image of a separate network tag annealed at 700 °C. The convex nanostructures keep the original morphology before 700 °C. Scale bars: 20 μm (**a**-**e**), 100 nm (**f**).

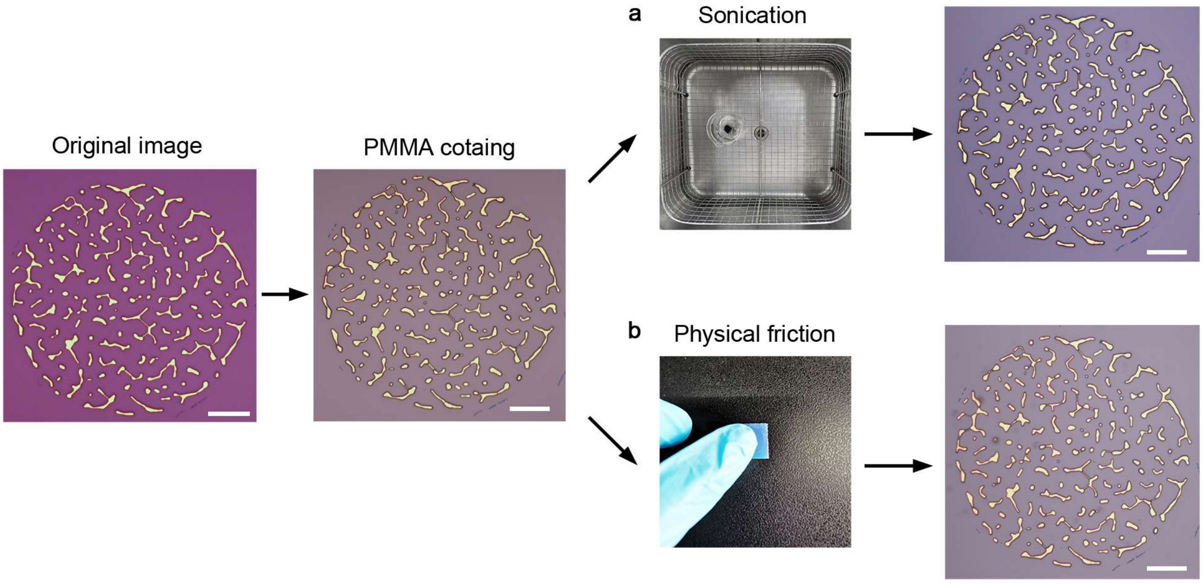

Supplementary Figure 16. Stability test under aqueous corrosion and mechanical friction. The network tag was first coated with a layer of PMMA and the physical features of the tag can still be recognized owing to the refractive index difference between Au and PMMA. **a** The label was sonicated in deionized water for 10 minutes. No changes of physical features can be observed. **b** The same label was rubbed on the coarse desktop repeating 10 times. No changes of physical features can be observed. Scale bars: 20 μm (**a** and **b**).

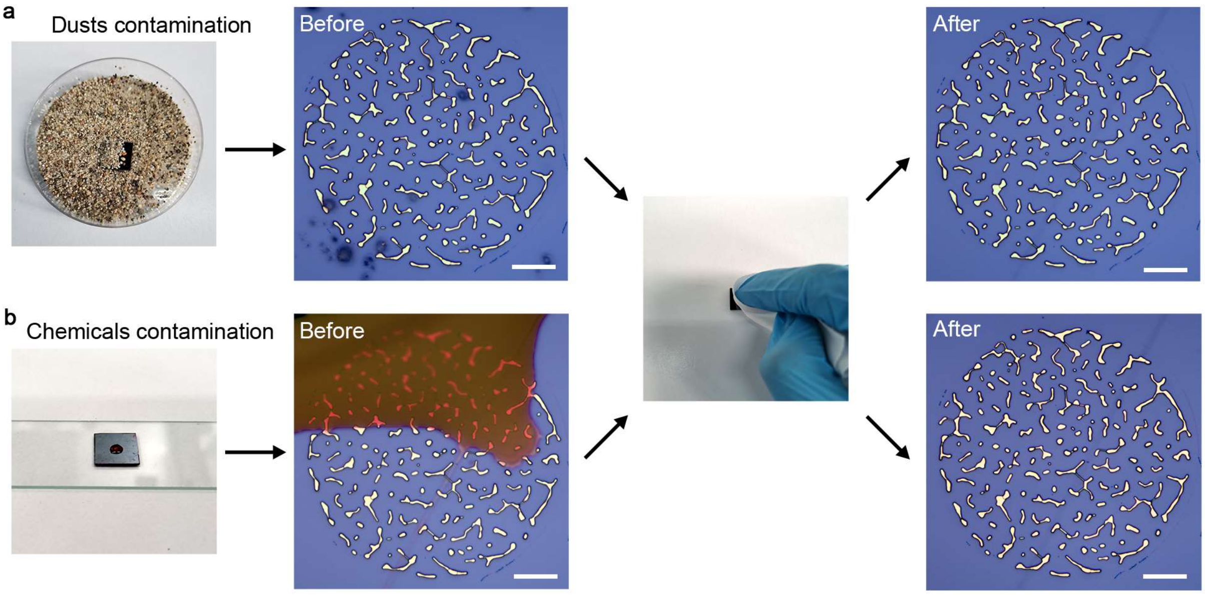

Supplementary Figure 17. Stability test under environmental contamination. a The label coated with PMMA was put into the sand and stirred repeatedly. In addition to a few introduced dusts (which can influence the tag identification), no changes of physical features can be observed. The label was then wiped with a soft cloth dipped in alcohol. Also, no changes of physical features can be observed, while the dusts have been completely removed. **b** The same label was exposed to the chemicals (rhodamine 6G). Parts of the network tag are covered by chemicals, which can also influence the tag identification. The label was then wiped with a soft cloth dipped in alcohol, and no changes of physical features can be observed, while the chemical stains have been completely removed. All the experiments verify that the PMMA protective layer can effectively prevent the network tag from being damaged in various real-world interference, meeting the demands of durability in practical application. Scale bars: 20 μm (**a** and **b**).

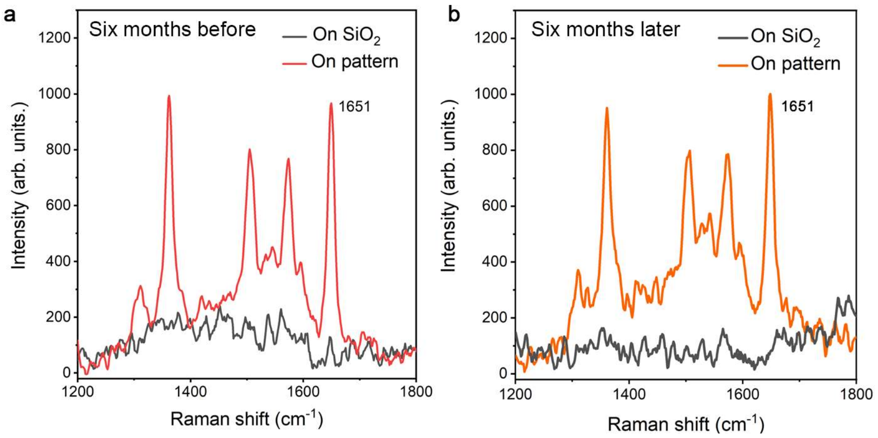

Supplementary Figure 18. Raman spectra of the same sample with the six-months interval, exhibiting almost no changes of the Raman intensity. This reveals that the chemical encoding can be kept stable for an extended period.

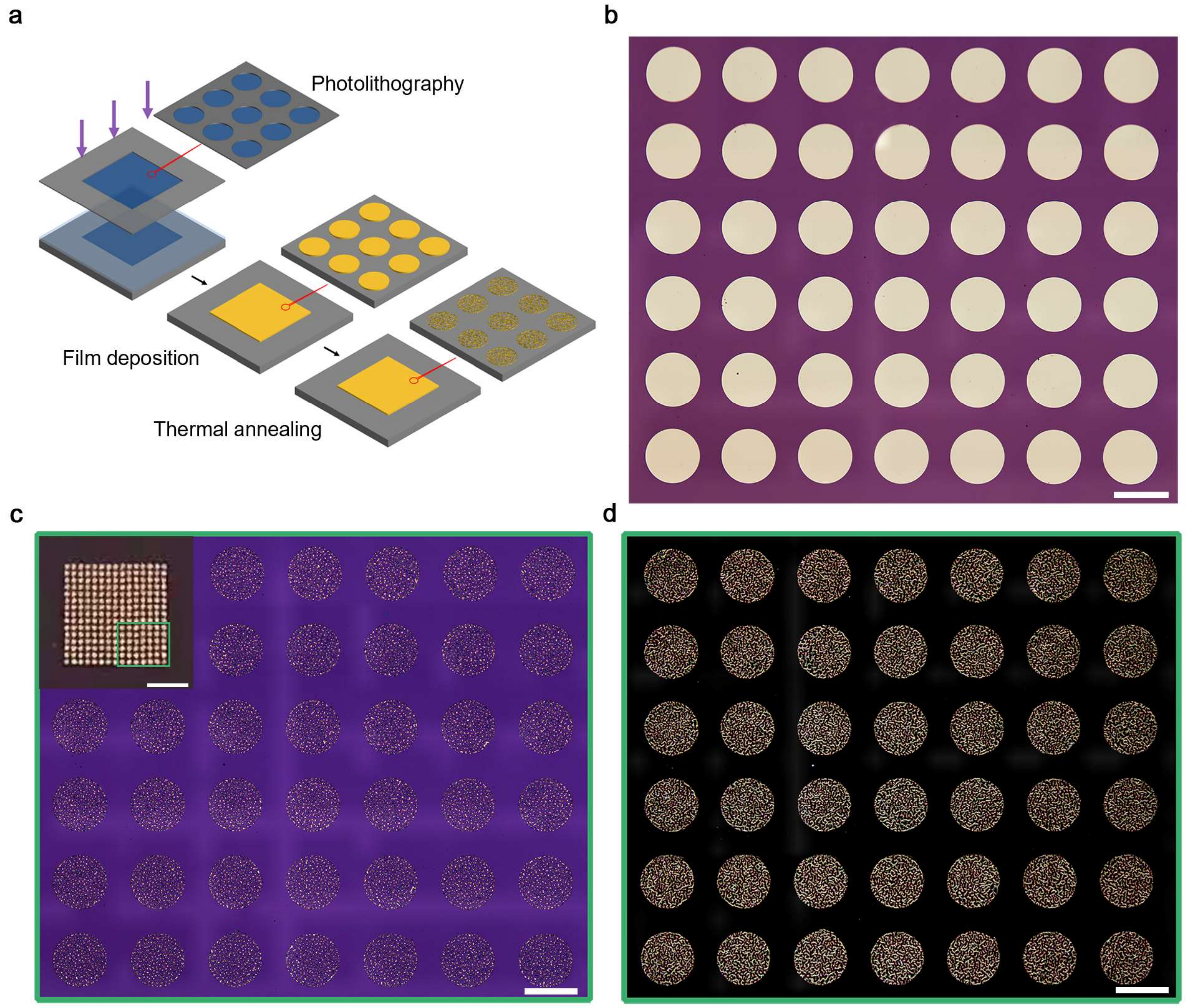

Supplementary Figure 19. Schematic illustration and Optical images presenting mass production of the PUF tags. a Schematic illustration of the production flow of the network tags, including the UV-photolithography, thin film deposition via magnetron sputtering, and one-step thermal annealing of the Au film. The fabrication process reveals the characteristics of mass production and scalability of the PUF tags. **b** Optical microscope image of the fabricated large-area Au film patterns after the magnetron sputtering. **c** Bright-field image of the Au network tags after the annealing process. The inset is the macro-scale graphic of the large-area network tags captured by the smartphone. **d** Dark-field image of the Au network tags. Scale bars: 120 µm (**b**-**d**), 1 mm (**c**, inset).

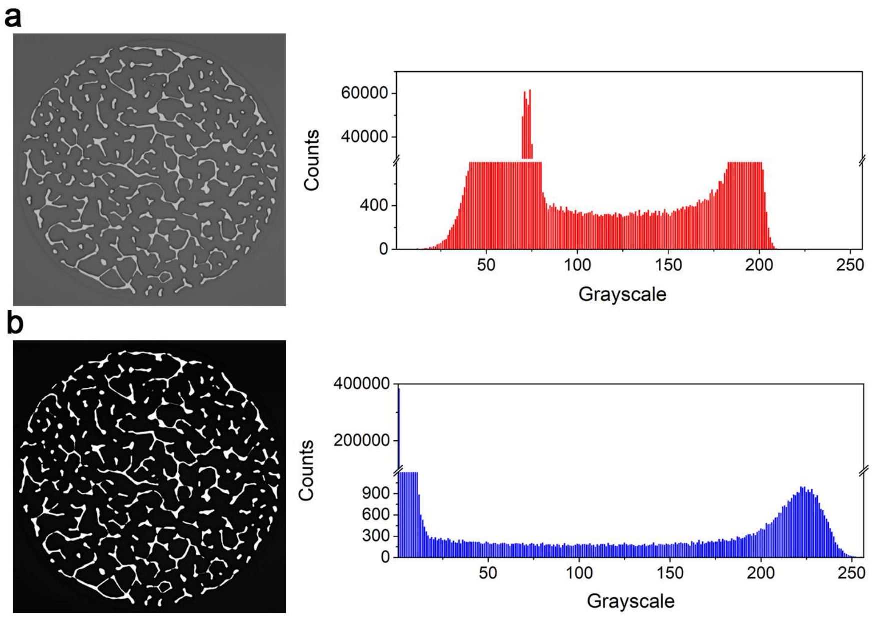

Supplementary Figure 20. The grayscale distribution before a and after b the image preprocessing of grayscale stretch. The grayscale stretch is used for unifying the image information, therefore, the influences of different conditions (brightness or contrast) can be suppressed in the deep learning model training.

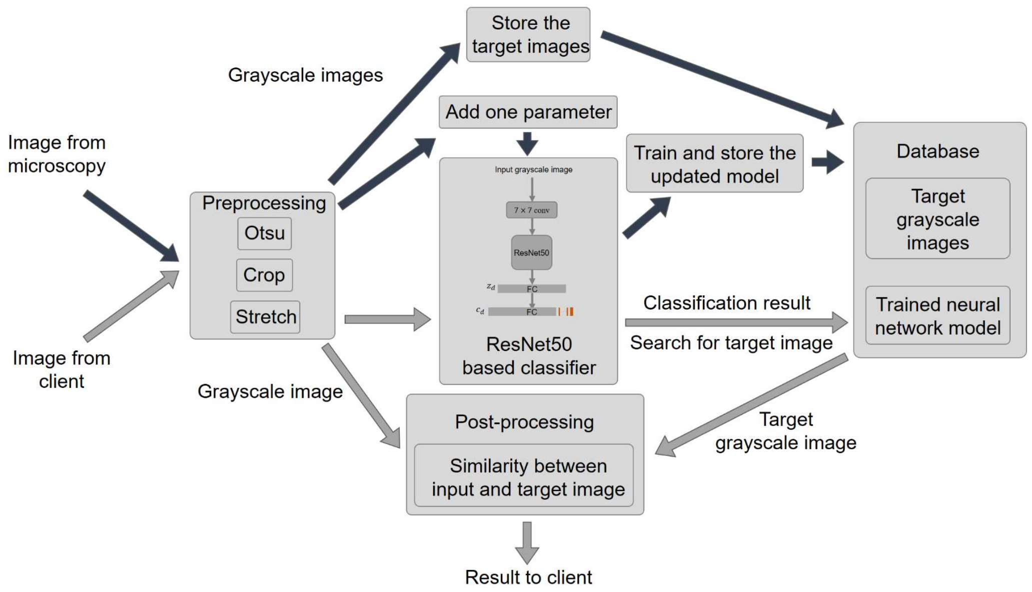

Supplementary Figure 21. The pipeline of the developed PUF key authentication software. This software includes two strategies: (i) add the new PUF pattern from microscopy to the database (STRATEGY I); (ii) search for the corresponding class of the input image from the client according to the database (STRATEGY II).

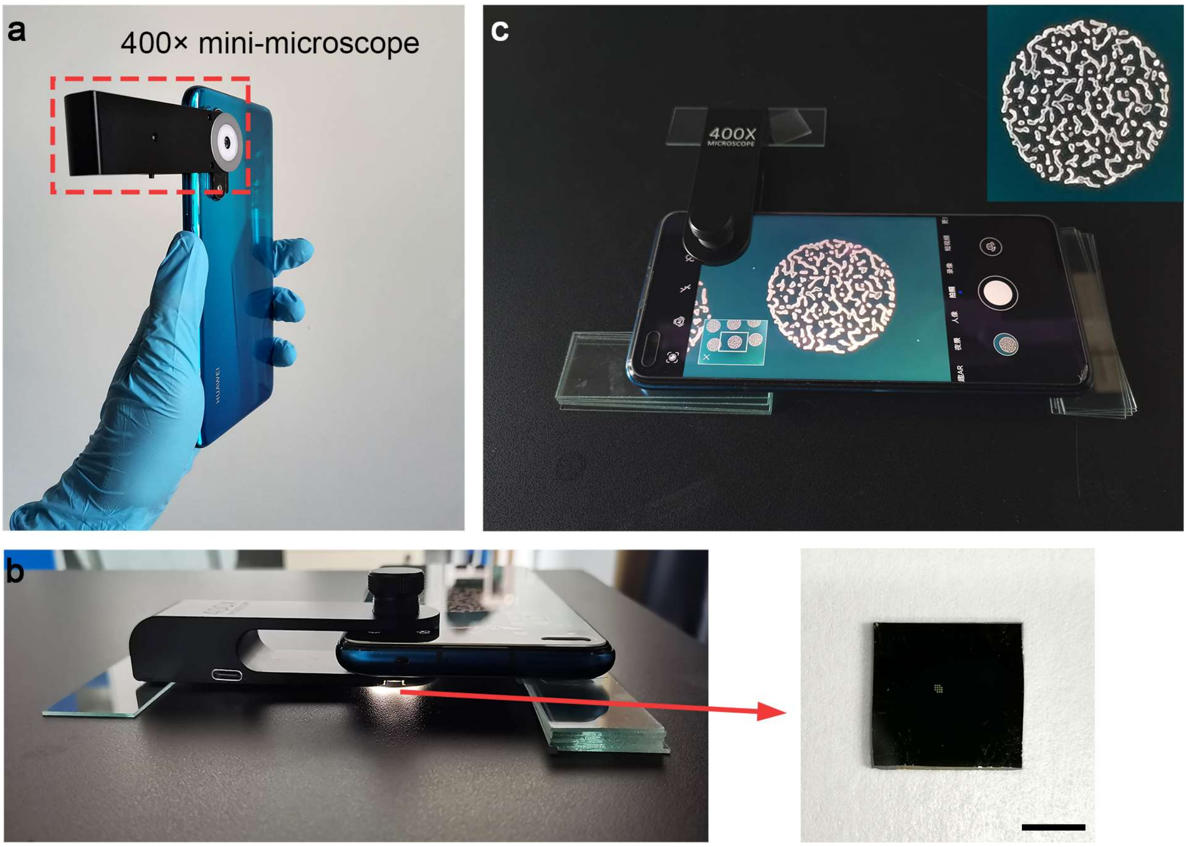

Supplementary Figure 22. Demonstration of PUF readout. a The readout tool of the smartphone connected with a 400× mini microscope (HUAWEI nova 6). **b** The smartphone was capturing the PUF pattern (Left) on the substrate (Right). **c** Top view of the readout process. The inset is the PUF pattern taken by the smartphone. Scale bar: 0.5 cm (**b**, right image).

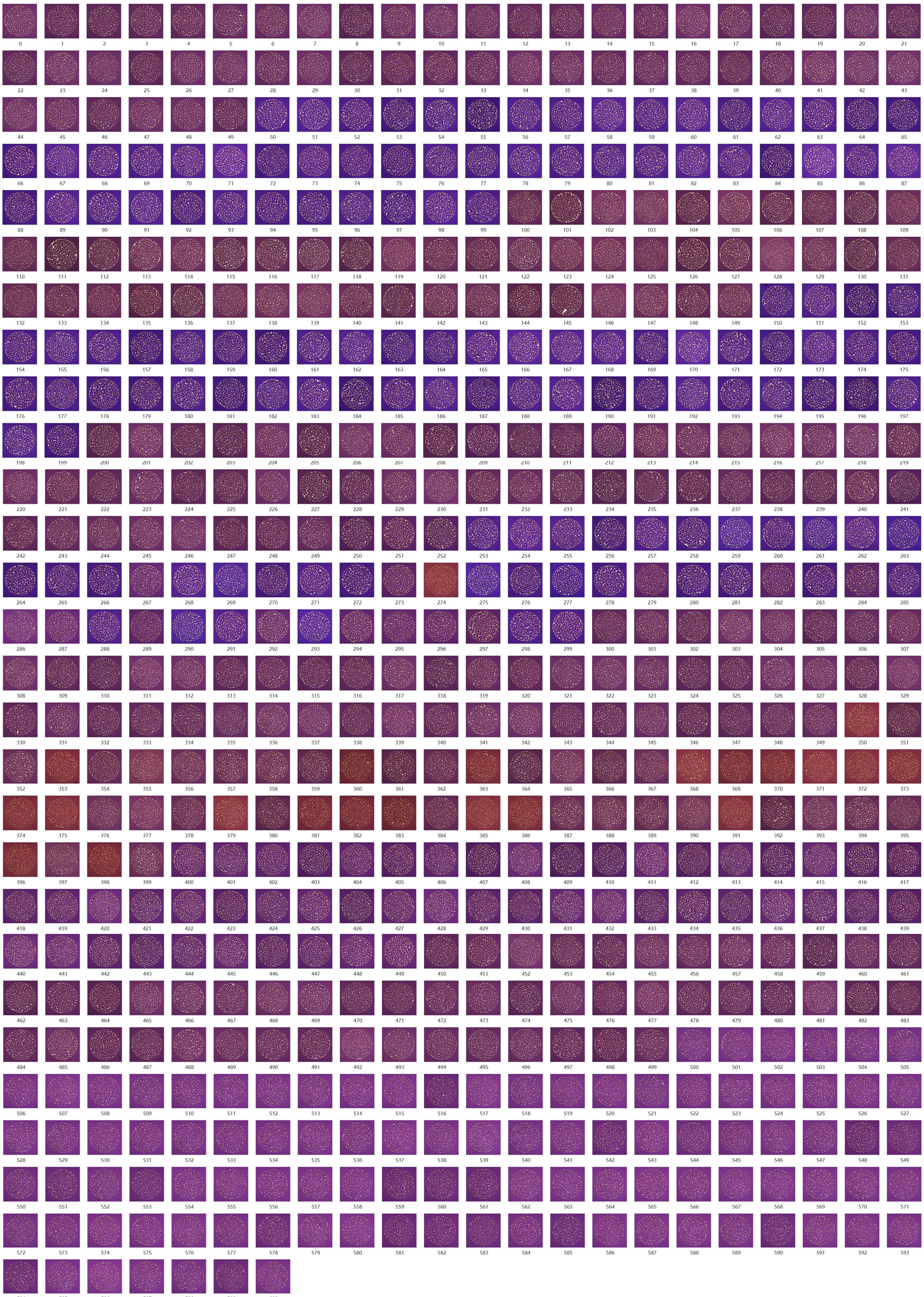
0 1 2 3 4 5 6 7 8 9 10 11 12 13 14 15 16 17 18 19 20 21
22 23 24 25 26 27 28 29 30 31 32 33 34 35 36 37 38 39 40 41 42 43
44 45 46 47 48 49 50 51 52 53 54 55 56 57 58 59 60 61 62 63 64 65
66 67 68 69 70 71 72 73 74 75 76 77 78 79 80 81 82 83 84 85 86 87
88 89 90 91 92 93 94 95 96 97 98 99 100 101 102 103 104 105 106 107 108 109
110 111 112 113 114 115 116 117 118 119 120 121 122 123 124 125 126 127 128 129 130 131
132 133 134 135 136 137 138 139 140 141 142 143 144 145 146 147 148 149 150 151 152 153
154 155 156 157 158 159 160 161 162 163 164 165 166 167 168 169 170 171 172 173 174 175
176 177 178 179 180 181 182 183 184 185 186 187 188 189 190 191 192 193 194 195 196 197
198 199 200 201 202 203 204 205 206 207 208 209 210 211 212 213 214 215 216 217 218 219
220 221 222 223 224 225 226 227 228 229 230 231 232 233 234 235 236 237 238 239 240 241
242 243 244 245 246 247 248 249 250 251 252 253 254 255 256 257 258 259 260 261 262 263
264 265 266 267 268 269 270 271 272 273 274 275 276 277 278 279 280 281 282 283 284 285
286 287 288 289 290 291 292 293 294 295 296 297 298 299 300 301 302 303 304 305 306 307
308 309 310 311 312 313 314 315 316 317 318 319 320 321 322 323 324 325 326 327 328 329
330 331 332 333 334 335 336 337 338 339 340 341 342 343 344 345 346 347 348 349 350 351
352 353 354 355 356 357 358 359 360 361 362 363 364 365 366 367 368 369 370 371 372 373
374 375 376 377 378 379 380 381 382 383 384 385 386 387 388 389 390 391 392 393 394 395
396 397 398 399 400 401 402 403 404 405 406 407 408 409 410 411 412 413 414 415 416 417
418 419 420 421 422 423 424 425 426 427 428 429 430 431 432 433 434 435 436 437 438 439
440 441 442 443 444 445 446 447 448 449 450 451 452 453 454 455 456 457 458 459 460 461
462 463 464 465 466 467 468 469 470 471 472 473 474 475 476 477 478 479 480 481 482 483
484 485 486 487 488 489 490 491 492 493 494 495 496 497 498 499 500 501 502 503 504 505
506 507 508 509 510 511 512 513 514 515 516 517 518 519 520 521 522 523 524 525 526 527
528 529 530 531 532 533 534 535 536 537 538 539 540 541 542 543 544 545 546 547 548 549
550 551 552 553 554 555 556 557 558 559 560 561 562 563 564 565 566 567 568 569 570 571
572 573 574 575 576 577 578 579 580 581 582 583 584 585 586 587 588 589 590 591 592 593
594 595 596 597 598 599 600

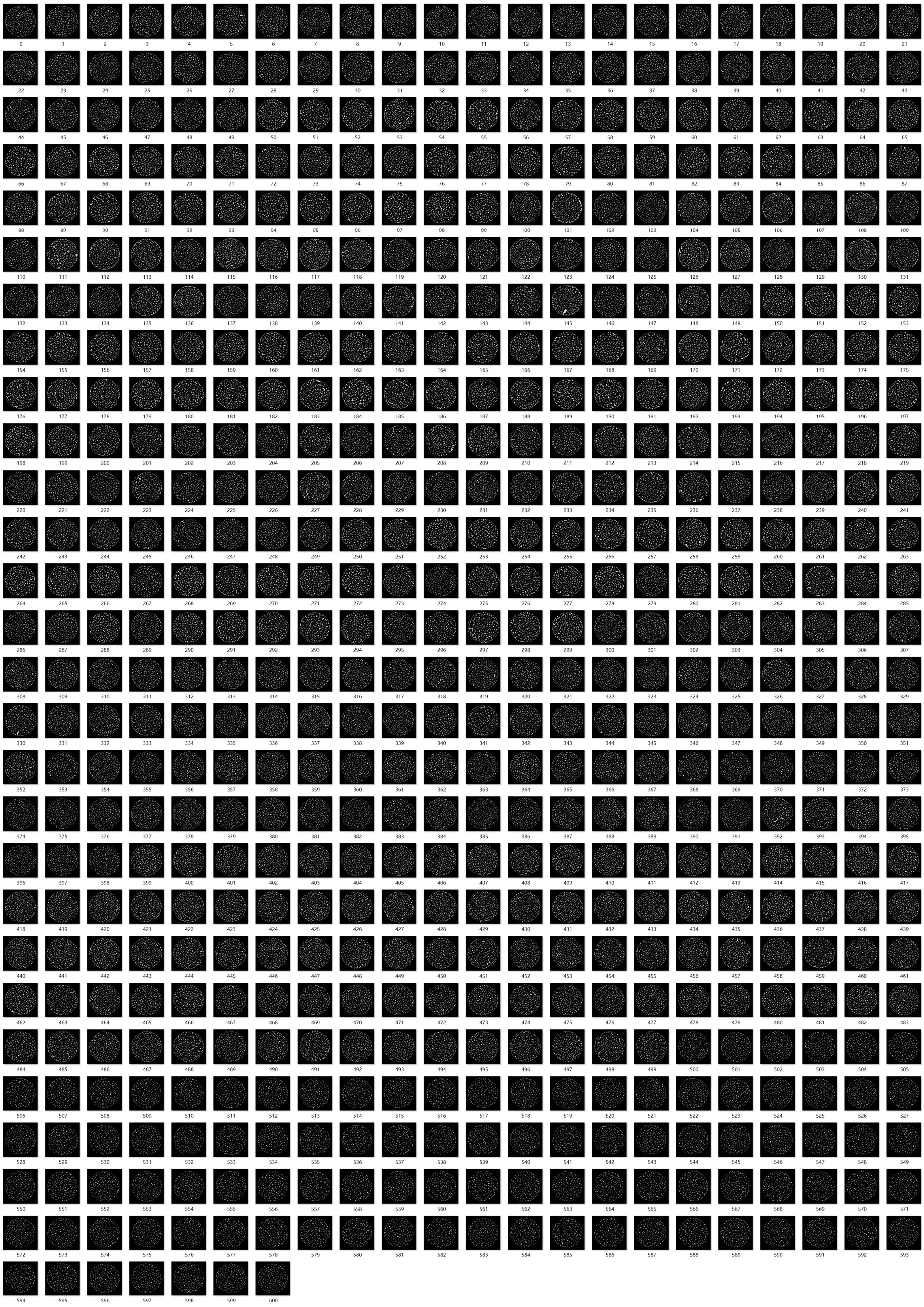

Supplementary Figure 23. The 600 images (RGB and corresponding grayscale images) as a part of the PUF key database for training/validation of the deep learning model. The 600 PUF patterns are also used for the calculation of the cross-correlation values to verify the tag uniqueness.

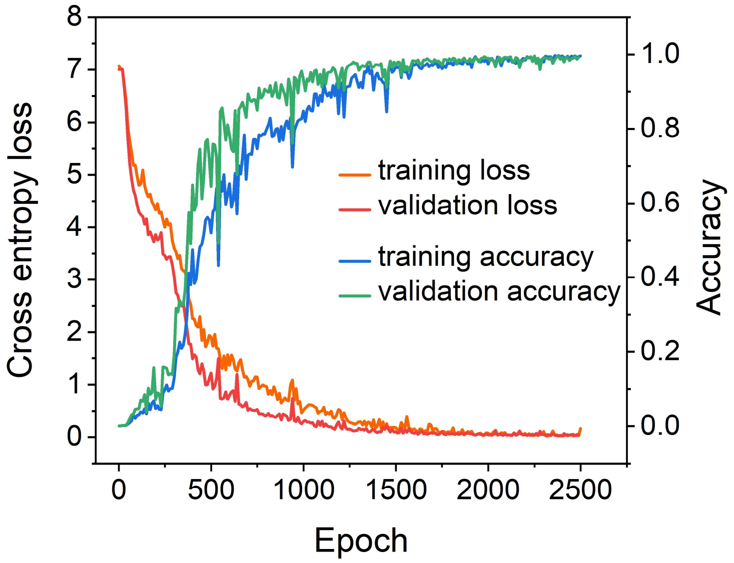

Supplementary Figure 24. Classification accuracy increased with CNN DL process. Training and test classification accuracies with increasing DL training cycles.

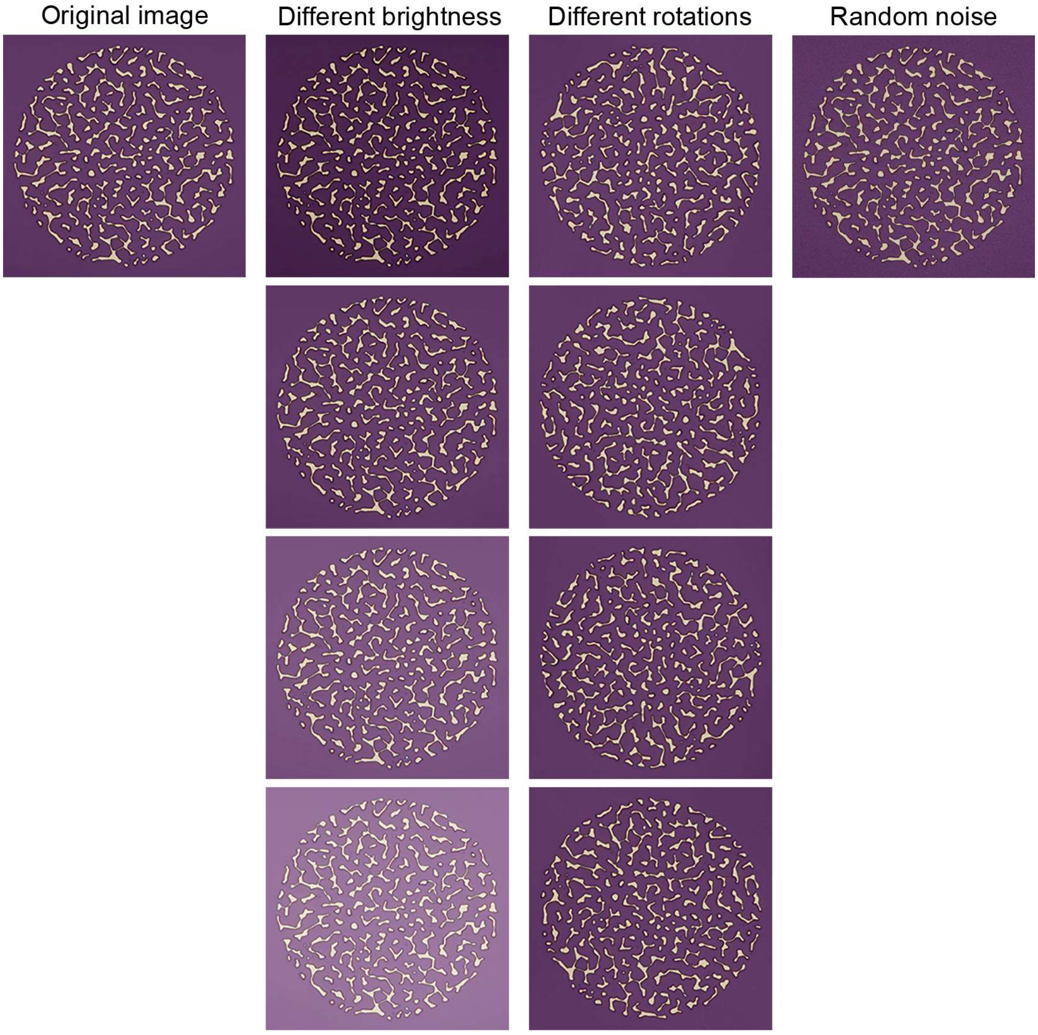

Supplementary Figure 25. Three kinds of images from the test-set with different brightness, rotation angles, and random noise for the validation of the established deep learning model.

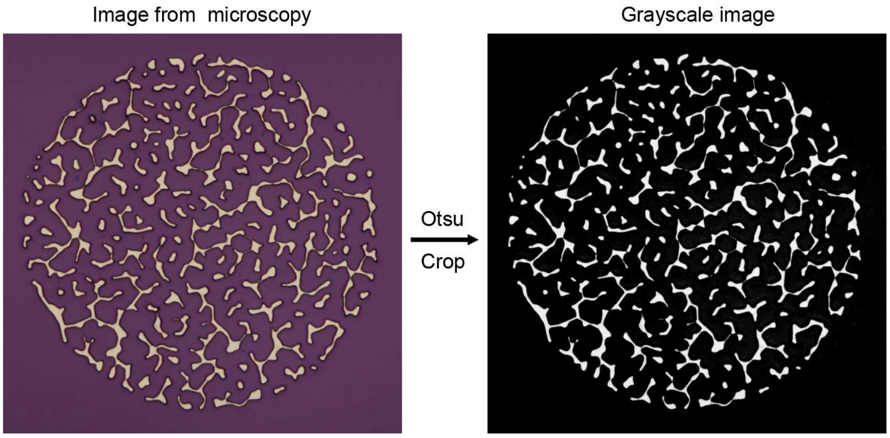

Supplementary Figure 26. The preprocessing of the uploaded image. The PUF image captured by the microscope is preprocessed by the localization of the PUF center (via an Otsu's method), crop, grayscale, and pixel distribution stretch.

Supplementary Table 1. The dataset information for the AI model training/validation/testing.

Dataset	Size	Augmentation	Labels
Original images (ORI_Set)	1850 PUFs		
Training set (TRAIN_Set)	1100 PUFs from ORI_Set	Rotation: 0, 30, 60, 90, .. , 330°	Corresponding classes: 0-1099
Validation set (VAL_Set)	The same PUFs with TRAIN_Set	Rotation: 1, 3, 5, 7, .. , 359°	Corresponding classes: 0-1099
Candidate set (CAND_Set)	200 PUFs from ORI_Set	Rotation: 0, 30, 60, 90, .. , 330° for training and 1, 3, 5, 7, .. , 359° for validation	Corresponding classes: 1100-1299
Fake set (FAKE_Set)	550 PUFs from ORI_Set	1) Rotation: 10 angles are randomly selected from those that are not considered in the TRAIN_Set and VAL_Set.<br>2) Gaussian noise for each image<br>3) 10 different brightness conditions applied to each image	All labelled as "FAKE"
Test set (TEST_Set)	1850 PUFs of ORI_Set with augmentations that generate 37000 images		Corresponding classes: 0-1299 for the first 1300 PUFs in the database, and "FAKE" for the left 550 PUFs

Supplementary References

1. Carro-Temboury, M. R., Arppe, R., Vosch, T. & Sorensen, T. J. An optical authentication system based on imaging of excitation-selected lanthanide luminescence. *Sci. Adv.* **4,** e1701384 (2018).
2. Arppe, R. & Sørensen, T. J. Physical unclonable functions generated through chemical methods for anti-counterfeiting. *Nat. Rev. Chem.* **1,** 0031 (2017).
